# A novel intubation discomfort score to predict painful unsedated colonoscopy

**DOI:** 10.1097/MD.0000000000024907

**Published:** 2021-03-12

**Authors:** Limei Wang, Hui Jia, Hui Luo, Xiaoyu Kang, Linhui Zhang, Xiangping Wang, Shaowei Yao, Qin Tao, Yanglin Pan, Xuegang Guo

**Affiliations:** a Xijing Hospital of Digestive Diseases, Air Force Medical University, 127 Changle West Road; b Shaanxi Second People's Hospital, 3 Shangqin Road, Xian; c Department of Digestive Diseases, Affiliated Hangzhou First People's Hospital, 261 Huansha Road, Hangzhou, China.

**Keywords:** colonoscopy score, pain, unsedated

## Abstract

Pain during colonoscopy is a critical quality indicator and often a limiting factor for unsedated colonoscopy. This study aimed to identify factors associated with pain during colonoscopy and establish a model for predicting a painful colonoscopy.

Patients aged 18 to 80 who underwent unsedated colonoscopy were prospectively enrolled in 2 tertiary endoscopic centers in China. The primary outcome was the rate of painful colonoscopy and then we identify high-risk factors associated with painful colonoscopy. A prediction model with an intubation discomfort score (IDS) was developed and validated.

Totally 607 patients participated in this study, including 345 in the training cohort and 262 in the validation cohort. Body mass index (BMI) of <18.5 kg/m^2^ (OR 2.18, 95% CI: 1.09–4.37), constipation (OR 2.45, 95% CI: 1.25–4.80), and anticipating moderate or severe pain (OR 2.06, 95% CI: 1.12–3.79) were identified as independent predictive factors for painful colonoscopy and used to develop the IDS (all *P* < .05). Patients with IDS ≥1 had increased insertion time [9.32(6.2–13.7)] minutes vs 6.87(5.1–10.4) minutes, *P* = .038) and decreased cecal intubation rate (96.0% vs 99.6%, *P* = .044). Abdominal compression (48.4% vs 19.9%, *P* < .001) and position change (59.7% vs 32.1%, *P* < .001) were more frequently required in the group of patients with IDS ≥1. These results were externally validated in a validation cohort.

The intubation discomfort score developed in this study was useful for predicting pain during colonoscopy, with IDS ≥1 indicating painful colonoscopy.

## 1 Introduction

Currently, colonoscopy is the standard method for the management of colorectal disease. Screening colonoscopy decreases the incidence and mortality of colorectal cancer by detection and treatment of precancerous lesions and early cancer.^[1,2]^ However, colonoscopy is regarded as relatively invasive, usually assumed to be an uncomfortable and sometimes painful procedure and associated with a low chance of completing the examination.^[3]^ Thus, sedated colonoscopy is recommended.^[4]^ However, several drawbacks of sedated colonoscopy have been reported, such as sedation-related complications, post-procedure activity restrictions, longer recovery time, requirement of an escort, and increased cost.^[5,6]^ These factors were positively associated with the unwillingness of patients to undergo colonoscopy. Hence, unsedated colonoscopy is gaining interest^[7]^ and has been recently advocated by several researchers.^[8–10]^

Previous studies have reported that 74% of patients felt no pain or only mild discomfort during unsedated colonoscopy.^[11]^ Thus, routine administration of sedative or analgesic agents to all patients was considered unnecessary. However, unsedated colonoscopy is considered an option for some but not for all patients.^[12]^ If we could identify patients who are at a high risk of experiencing pain during colonoscopy at the preoperative stage, targeted administration of sedatives or special techniques could be recommended.

Here, we prospectively collected the data of patients undergoing colonoscopy with an aim to investigate the possible risk factors associated with painful colonoscopy using a stepwise multivariate regression model. Furthermore, we developed a novel point score to predict whether patients were at a high risk of a painful colonoscopy so that another appropriate method of colonoscopy or the aid of special techniques can be recommended to facilitate the completion of the procedure.

## 2 Patients and method

### 2.1 Patients

This prospective study was conducted at 2 tertiary centers in China. The patients of the training cohort were enrolled from the Xijing Hospital of Digestive Diseases; those of the validation cohort were enrolled from the Shaanxi Second People's Hospital. The study protocol were approved by the ethics committee of Xijing Hospital and Shaanxi Second People's Hospital. Written informed consent was obtained from all patients.

Consecutive patients aged 18 to 80 years old who willing to undergo unsedated colonoscopy were enrolled in this study. Exclusion criteria included the following: no bowel preparation or colon cleansing by enema only; no need to reach the cecum; prior finding of severe colon stenosis or obstructing tumor; history of colectomy, unstable hemodynamics; pregnant or breastfeeding women; and inability to provide informed consent. Both training and validation cohorts were enrolled with the same inclusion and exclusion criteria. This study was registered with ClinicalTrials.gov (NCT03540173).

### 2.2 Bowel preparation and unsedated colonoscopy

All patients were prescribed polyethylene glycol electrolyte powder (PEG-4000e; Wanhe Pharmaceutical Co, Shenzhen, China) for bowel preparation according to the preference of the treating physician. The patients were asked to drink the first 2 L of PEG4000e between 19:00 and 20:00 on the night before the colonoscopy within 2 hours. Subsequently, on the day of the examination and 5 hours before the procedure, the patients were asked to consume the remaining 2 L. Patients were encouraged to drink more clear liquids after purgatives for adequate hydration before colonoscopy. In addition, they were instructed to have a regular meal for lunch and only liquid diet for dinner on the day before the operation. This preparation method has been previously reported with an acceptable cleansing rate.^[11]^

All colonoscopies were performed between 08:00 and 13:00 by 4 experienced colonoscopists. Before the start of the study, all the colonoscopists had performed >3000 colonoscopies independently. A high-resolution adult video colonoscope (EC-590WM; Fujinon, Japan) was used for every procedure.

### 2.3 Data collection and outcome measurement

The following variables were systematically collected: demographic data [age, sex, weight, height, body mass index (BMI), level of education, and marital status]; indication for colonoscopy (screening, surveillance, and diagnosis); and medical history (smoking, alcohol consumption, constipation (defined by the Rome IV diagnostic criteria),^[13]^ surgery, and comorbidities). Anxiety status was evaluated using the hospital anxiety and depression (HAD) scale.^[14]^ Abdominal pain during the examination was evaluated by a previously validated 4-point verbal rating scale (no, slight, moderate, and severe pain),^[11]^ and moderate or severe pain during the procedure was assumed to indicate “painful colonoscopy;” the degree of pain was recorded in all patients with complete and incomplete colonoscopy. The patients were also asked to grade their anticipated pain and normal abdominal pain using the same 4-point verbal rating scale before the examination. All data were collected by 1 investigator (WLM) who did not participate in the data analysis.

### 2.4 Statistical analysis

Sample size calculation was performed by maximum likelihood estimation based on logistic regression as described previously.^[15]^ In the current study, events per variable were set as 27 according to our previous experience. The number of possible predictors were 11 in this study. Thus, about 300 patients may be sufficient to produce significant power to identify risk factors associated with pain. To compensate the possible drop-out, 330 patients were planned to be enrolled in the training cohort.

The training cohort was used to determine the factors influencing pain during colonoscopy and to develop the IDS, and the validation cohort was used to verify the IDS. Categorical variables were described as percentages. Continuous variables were described as means ± standard deviation (SD) or medium (range). Chi-Squared test was used comparing categorical variables when appropriate. Student *t* test or one-way ANOVA was used comparing normal distributed continuous variables. To assess the factors associated with painful colonoscopy, a multivariate logistic regression analysis was performed using the variables with *P* values <.1 in the univariate analysis. The cutoff values of the quantitative variables and IDS were determined by receiver operator characteristics (ROC) analysis.

All tests of significance were two-tailed, and *P* < .05 was considered statistically significant. Analyses were mainly performed with SPSS version 19.0 (IBM Corp, Armonk, NY). Subgroup analyses were performed with Stata 12.0 (StataCorp, College Station, TX).

## 3 Results

### 3.1 Baseline patient characteristics

**Figure 1:**
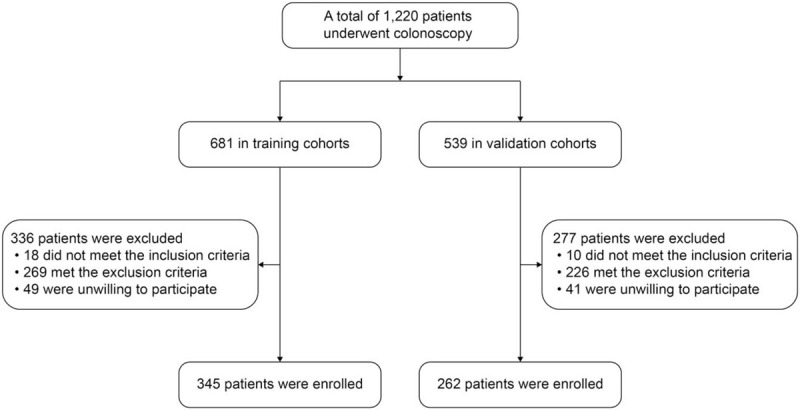
Study flowchart.

A total of 1220 patients undergoing unsedated colonoscopy from March 2018 to October 2018 were prospectively enrolled in this study (681 in the training and 539 in the validation cohort), of which 613 were excluded (18 did not meet the inclusion criteria, 269 met the exclusion criteria, and 49 were unwilling to participate in the training cohort and 10 did not meet the inclusion criteria, 226 met the exclusion criteria, and 41 were unwilling to participate in the validation cohort). Finally, 345 patients from the Xijing Hospital of Digestive Diseases and 262 patients form Shaanxi Second People's Hospital were included in this study (Fig. 1).

Table 1 shows the baseline demographics of 2 cohorts. Majority of the colonoscopies were performed for diagnosis (79.7% and 74.8% in the training and validation cohorts, respectively); 92 (26.7%) and 62 (23.7%) of the patients reported moderate or severe pain before colonoscopy in the training and validation cohorts, respectively. Expectation of Painful colonoscopy was noted in 62 (18.3%) and 47 (17.9%) patients in the training and validation cohorts, respectively. No complications were noted in both cohorts. In both cohorts, almost two-thirds of patients (76.8% in the training cohort and 77.1% in the validation cohort) received high school or above education. Tricyclic antipsychotics (TCA) and narcotics were taken in 6 (1.7%) and 1 (0.3%) of patients respectively in the training cohort. The number was 4 (1.5%) and 2 (0.8%) in the validation cohort.

**Table 1:** Patient characteristics.

	Training cohort (n = 345)	Validation cohort (n = 262)	*P* value
Age (years)^∗^	49.3 ± 14.5	51.3 ± 13.0	.077
Males (%)	174 (50.4%)	139 (53.1%)	.523
BMI (kg/m^2^)^∗^	22.3 ± 3.7	22.4 ± 3.8	.761
Grade of education (%)			.934
Elementary school or no education	80 (23.2%)	60 (22.9%)	
Higher than elementary school	265 (76.8%)	202 (77.1%)	
Marriage status (%)			.506
Single	23 (6.7%)	14 (5.3%)	
Married	322 (93.3%)	248 (94.7%)	
Smoking (%)	74 (21.5%)	52 (19.9%)	.630
Drinking (%)	79 (22.9%)	57 (21.8%)	.738
Medicines (%)			
TCA	6 (1.7%)	4 (1.5%)	.893
Narcotics	1 (0.3%)	2 (0.8%)	.811
Others	27 (7.8%)	21 (8.0%)	.932
HAD Scale (%)			.653
<8	293 (84.9%)	219 (83.6%)	
≥8	52 (15.1%)	43 (16.4%)	
Previous surgery (%) (pelvic or abdominal)	100 (29.0%)	62 (23.7%)	.142
Minimally invasive surgery	53 (15.4%)	37 (14.1%)	.670
Colonoscopy indication (%)			.356
Screening	11 (3.2%)	10 (3.8%)	
Surveillance	59 (17.1%)	56 (21.4%)	
Diagnostic	275 (79.7%)	196 (74.8%)	
Main symptoms (%)			.424
Abdominal pain	100 (29.0%)	72 (27.5%)	
Diarrhea	62 (18.0%)	36 (13.7%)	
Distention	39 (11.3%)	28 (10.7%)	
Constipation	46 (13.3%)	39 (14.9%)	
Melena	19 (5.5%)	24 (9.2%)	
Others	79 (22.9%)	63 (24.1%)	
Normal abdominal pain (%)			.400
No and slight	253 (73.3%)	200 (76.3%)	
Moderate and severe	92 (26.7%)	62 (23.7%)	
Expectation of pain (%)			.919
No and slight	282 (81.7%)	215 (82.1%)	
Moderate and severe	63 (18.3%)	47 (17.9%)	

BMI = body mass index, HAD = hospital anxiety and depression scale.

∗ Values are mean ± standard deviation.

### 3.2 Regression analysis and IDS calculation in the training cohort

In the training cohort, we evaluated the influence of patient-related factors on pain during colonoscopy. Univariate logistic regression analysis indicated that age, marital status, BMI, constipation, and expectation of pain before colonoscopy may influence the degree of pain during the examination. After multivariate logistic regression analysis, only low BMI (OR 2.18, 95% CI 1.09–4.37), constipation (OR 2.45, 95% CI 1.25–4.80), and anticipation of moderate or severe pain (OR 2.06, 95% CI 1.12–3.79) had a significant influence on painful colonoscopy (Table 2). In the development of the model for the prediction of painful colonoscopy, each factor related to painful colonoscopy was given 1 point. Thus, the IDS = 1 × B (1 if BMI <18.5 kg/m^2^, 0 if ≥18.5 kg/m^2^) +1 × C (1 if constipation is present, 0 if not) +1 × E (1 if moderate or severe pain was expected, 0 if not or only slight pain was expected).

**Table 2:** Univariate and multivariate analyses of factors associated with painful colonoscopy in the training cohort.

	Univariate analysis	Multivariate analysis		
	OR (95% CI)	*P* value	OR (95% CI)	*P* value
Age (years)				
≥50	1		1	
<50	1.52 (0.93–2.48)	.092	1.46 (0.84–2.53)	.179
Sex				
Female	1			
Male	0.68 (0.42–1.11)	.120		
Marital status				
Married	1		1	
Single	2.25 (0.95–5.32)	.065	1.36 (0.51–3.59)	.538
BMI (kg/m^2^)				
≥18.5	1		1	
<18.5	2.96 (1.55–5.66)	.001	2.18 (1.09–4.37)	.028
Constipation				
No	1		1	
Yes	2.70 (1.43–5.11)	.002	2.45 (1.25–4.80)	.009
Colonoscopy indication				
Screening	1			
Surveillance	1.57 (0.33–7.46)	.568		
Diagnostic	0.80 (0.43–1.48)	.470		
Previous surgery (pelvic or abdominal)				
Yes	1			
No	0.98 (0.58–1.65)	.929		
HAD scale				
<8	1			
≥8	1.02 (0.52–1.98)	.964		
Level of education				
Elementary school or no education	1			
Higher than elementary school	0.78 (0.47–1.32)	.356		
Normal abdominal pain				
No and slight	1			
Moderate and severe	1.29 (0.80–2.09)	.295		
Expectation of pain				
No and slight	1		1	
Moderate and severe	2.50 (1.42–4.43)	.002	2.06 (1.12–3.79)	.021

BMI = body mass index, HAD = hospital anxiety and depression.

### 3.3 Prediction of painful colonoscopy in the training and validation cohorts using IDS

We calculated and verified the IDS in the training and validation cohorts. The IDS identified groups with distinct outcomes in both cohorts; an increased IDS indicates a more intense pain during colonoscopy (Table 3).

**Table 3:** Painful colonoscopy rate with different IDS.

		Training Cohort (n = 345)				Validation cohort (n = 262)				
IDS	0	1	2	3	*P* Value	0	1	2	3	*P* Value
Pain rate	69/221 31.2%	53/82 64.6%	26/37 70.3%	4/5 80.0%	<.001	44/145 30.3%	51/79 64.6%	25/32 78.1%	5/6 83.3%	<.001

IDS = Intubation Discomfort Score.

**Figure 2:**
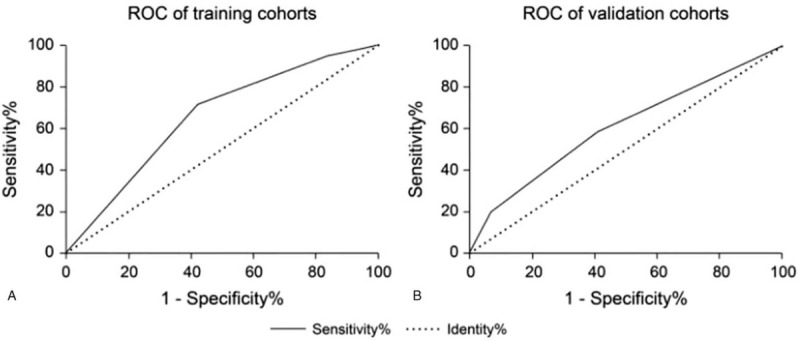
(A) ROC curve of IDS for the prediction of painful colonoscopy in the training cohort. (B) ROC curve of IDS for the prediction of painful colonoscopy in the validation cohort.

Based on the IDS, patients could be classified as low-risk (IDS < 1) and high-risk (IDS ≥ 1) groups by ROC curve analysis. The area under the ROC curve of the IDS for the prediction of painful colonoscopy was 0.66 (95% CI 0.59–0.72) and 0.61 (95% CI 0.53–0.69) in the training and validation cohorts, respectively (Fig. 2A, B), with an optimal threshold of 1 point. Sensitivity, specificity, positive predictive value, and negative predictive value of IDS ≥ 1 for predicting painful colonoscopy were, respectively, 57.6%, 71.9%, 42.7%, and 82.4% in the training cohort and 58.1%, 40.5%, 30.8%, and 82.1% in the validation cohort. We further analyzed the difference in insertion time, cecal intubation rate, the need for abdominal compression, and position changes in patients with different IDS (Table 4). Patients with IDS ≥ 1 had a significantly longer insertion time than those with IDS<1 both in the training and validation cohorts. Insertion time in low-risk patients was 6.87 (5.1–10.4) minutes and 6.57 (4.9–11.0) minutes in the training and validation cohorts, respectively. By contrast, high-risk patients had a longer insertion time [training cohort, 9.32 (6.2–13.7) minutes; validation cohort, 9.68 (6.9–14.1) minutes]. The cecal intubation rate in patients with IDS < 1 was greater than that in patients with IDS ≥ 1 both in the training (99.6% vs 96.0%) and validation (99.3% vs 94.9%) cohorts. We also evaluated the need for abdominal compression and position changes during colonoscopy and found that abdominal compression and position changes are more often needed in high-risk patients in both cohorts. Besides, we collected information on the willingness of patients to repeat the unsedated colonoscopy. A total of 18 high-risk patients refused to repeat unsedated colonoscopy.

**Table 4:** Effect of IDS on colonoscopy procedure.

	Training cohort (n = 345)			Validation cohort (n = 262)		
	IDS≥1 (n = 124)	IDS<1 (n = 221)	*P* value	IDS≥1 (n = 117)	IDS<1 (n = 145)	*P* value
Painful colonoscopy rate (%)	83 (66.9%)	69 (31.2%)	<.001	81 (69.2%)	44 (30.3%)	<.001
Insertion time^∗^ (minute)	9.32 (6.2-13.7)	6.87 (5.1–10.4)	.038	9.68 (6.9–14.1)	6.57 (4.9–11.0)	.021
Cecal intubation rate (%)	119 (96.0%)	220 (99.6%)	.044	111 (94.9%)	144 (99.3%)	.067
Abdominal compression (%)	60 (48.4%)	44 (19.9%)	<.001	57 (48.7%)	33 (22.8%)	<.001
Position change (%)	74 (59.7%)	71 (32.1%)	<.001	70 (59.8%)	54 (37.2%)	<.001

IDS = intubation discomfort score.

∗ Values were expressed as medium (range).

In order to investigate whether IDS score is still useful after excluding the patients undergoing diagnostic colonoscopy, we did a sensitivity analysis to investigate in both training and validation cohort, the rates of painful colonoscopy significantly elevated when IDS increased from 0 to 3 (Supplementary Table 1). Similarly, patients with IDS ≥ 1 had higher rate of painful colonoscopy, longer insertion time, and more frequent requirement of abdominal compression and position change (Supplementary Table 2). All of these results indicated that IDS score is also useful for patients undergoing screening or surveillance colonoscopy.

We also compared the HAD scores between patients undergoing diagnostic and screening/surveillance colonoscopy. There were no significant difference between the 2 groups [0 (0–4) vs 0 (0–3)] in training cohort, *P* = .338; 0 (0–3) vs 0 (0–4) in validation cohort, *P* = .709). HAD scores was not found to be correlated or associated with the pain score in this study (Supplementary Table 3).

## 4 Discussion

Unsedated colonoscopy has some advantages, and nearly 80% of the patients could undergo unsedated colonoscopy with mild or no pain.^[10,12]^However, approximately 20% of patients experience pain during intubation, which may in turn prevent patients from participating in screening colonoscopy;^[16]^ thus, identifying patients who are more likely to experience pain is essential before colonoscopy. In this study, we found that low BMI (<18.5 kg/m^2^), constipation, and anticipation of a painful procedure are independent factors associated with painful colonoscopy, and we also showed that the insertion time was significantly longer in high-risk patients; these results are consistent with those of a previous study.^[17]^ Moreover, in our study, we developed a simple scoring system (IDS ≥ 1 for high-risk patients) to identify those who are likely to be at high risk of experiencing pain during colonoscopy, which could in turn help in choosing or recommending for painless colonoscopy and other special examination methods.

In slender patients, angulation of the sigmoid colon may be sharper and may require constant straightening, which contributes to painful colonoscopy. Our study used BMI as an indicator of obesity and found that a low BMI is associated with painful colonoscopy; this result is consistent with that in a previously published research.^[6]^ However, BMI alone is not a suitable representative of abdominal or visceral fat. Thus, further studies should evaluate waist or hip circumference as a predictor of painful colonoscopy. Moreover, we also found that anticipation of pain among patients is correlated with painful colonoscopy, which was also reported previously.^[10]^ While constipation as a factor has not been mentioned in previous research, patients with irritable bowel syndrome have been reported to experience more pain;^[18,19]^ some patients in our study had irritable bowel syndrome. Furthermore, several studies have demonstrated that nervousness or anxiety before the examination contributes to a painful colonoscopy.^[20]^ However, in our study, the HAD scale had no correlation with painful colonoscopy, which could be because the HAD scale is a tool to evaluate normal anxiety level and thus may not reflect the level of anxiety regarding the procedure. In addition, some researchers believed that a history of abdominal or pelvic surgery could lead to a more difficult and painful colonoscopy.^[7,21]^ By contrast, such observation was not noted in approximately 60% of the patients who had a previous surgery, which could be attributed to the minimally invasive surgery they underwent. Female sex and younger age were considered risk factors for painful colonoscopy.^[22,23]^ Although no statistically significant correlation between these 2 factors and painful colonoscopy in our study was observed, we found that women and younger patients tend to experience more discomfort.

We established a simple and easy scoring system to classify patients into high- and low-risk groups, which could in turn aid in the selection of the most appropriate method for colonoscopy. Patients with low-risk scores had significantly shorter insertion time and less abdominal compression and position change. This phenomenon suggested that the procedure of intubation seemed to be easier in patients with less pain. For patients at low risk (IDS < 1), unsedated colonoscopy may be suitable. For patients at high risk of experience pain (IDS ≥ 1), there are several methods which could be useful for preventing or reducing pain. Firstly, it has been reported water exchange method instead of air insufflation can reduce pain during intubation in several high-quality studies.^[24,25]^ This technique can be tried in unsedated patients with IDS > 1. Secondly, CO2 insufflation, the use of small-caliber endoscopes or cap-assisted colonoscopy^[26]^ may be helpful to alleviate pain. Lastly, sedation can ensure patients painless during the whole procedure.

The strength of this study is its design. First, the relevant data of non-anesthetic colonoscopy were collected prospectively, and the pain prediction model was established by evaluating the factors affecting the degree of pain during colonoscopy in the experimental group. A validation group was set up to validate the result of the experimental group. Second, the scoring system developed was simple to it use facilitate among clinical workers. Lastly, the multicenter design of the study makes the results more generalizable.

Nevertheless, this study has some limitations. Firstly, the experience of endoscopists may significantly affect the pain associated with the procedure and the willingness of patients to repeat the examination. In the present study, all colonoscopies were performed by experienced endoscopists who had performed >3000 colonoscopies independently. The findings based on the performance of experienced endoscopists may not be generalized to the procedures performed by trainees or inexperienced endoscopists. Secondly, although the sensitivity of IDS score was suboptimal, negative predictive value was more than 80% in both training and validation cohorts, which meant most of patients with IDS < 1 (without any high-risk factors) would be suitable for unsedated colonoscopy. In previous studies, there was not better prediction model to predict painful colonoscopy. The efficiency of the model may be further improved by collecting more patient-related parameters or enrolling more patients in further clinical trials. Thirdly, the current study only enrolled unseated patients, the number of these patients is limited since most of patients underwent sedated colonoscopy in clinical practice. The number of unsedated patients included in this study seems relatively little. Some risk factors associated with pain may be missed due to the small sample size and patients willing to undergo unsedated colonoscopy were enrolled in this study would result in underestimation of the real risk of painful colonoscopy in the whole population, which could be further investigated in larger studies with involvement of more endoscopic centers. Last but not least, psychotropic or narcotic drugs can increase the pain threshold during colonoscopy. Only less than 3% of patients in this study took TCA or narcotics. No significant differences were found between patients taking TCA or narcotics and those not taking. However, the effects of TCA or narcotics on painful feeling during colonoscopy deserves further investigation in larger studies.

In summary, a low BMI (<18 kg/m^2^), constipation, and anticipation of pain are associated with painful colonoscopy, and we developed a novel, objective, noninvasive, and conveniently applicable scoring system to predict painful colonoscopy in the preoperative stage.

## Author contributions

Yanglin Pan, Xuegang Guo participated in the design of the study; Limei Wang, Shaowei Yao, Qin Tao participated in the data collection and the design of the study; Xiaoyu Kang, Hui Luo participated in the analysis and manuscript preparation; Limei Wang, Hui Jia, Hui Luo participated in the data analyses and wrote the manuscript; Linhui Zhang, Xiangping Wang give critical revision of the manuscript for important intellectual content.

## Supplementary Material

Supplemental Digital Content

## Supplementary Material

Supplemental Digital Content

## Supplementary Material

Supplemental Digital Content
