# Supplementary material for: A novel intubation discomfort score to predict painful unsedated colonoscopy
Source: Medicine (Baltimore). 2021 Mar 12;100(10):e24907. doi: 10.1097/MD.0000000000024907 (PMC7969226; doi:10.1097/MD.0000000000024907)
Supplement: Supplemental Digital Content [file medi-100-e24907-s001.docx]

**Supplementary Table 1.** Painful colonoscopy rate with different IDS

(excluding diagnostic patients)

|  |  | Training Cohort (n=71) | | | | Validation Cohort (n=66) | | | | |
| --- | --- | --- | --- | --- | --- | --- | --- | --- | --- | --- |
| IDS | 0 | 1 | 2 | 3 | PValue | 0 | 1 | 2 | 3 | PValue |
| Pain rate | 7/43  16.3% | 6/19  31.6% | 4/6  66.7% | 3/3  100.0% | 0.002 | 5/35  14.3% | 7/19  36.8% | 5/8  62.5% | 3/4  75.0% | 0.006 |

IDS, Intubation Discomfort Score
