# Supplementary material for: A novel intubation discomfort score to predict painful unsedated colonoscopy
Source: Medicine (Baltimore). 2021 Mar 12;100(10):e24907. doi: 10.1097/MD.0000000000024907 (PMC7969226; doi:10.1097/MD.0000000000024907)
Supplement: Supplemental Digital Content [file medi-100-e24907-s002.docx]

**Supplementary Table 2.** Effect of IDS on colonoscopy procedure

(excluding diagnostic patients)

|  | Training cohort (n=71) | | | | Validation cohort (n=66) | | |
| --- | --- | --- | --- | --- | --- | --- | --- |
|  | IDS≥1 (n=28) | IDS<1 (n=43) | | p value | IDS≥1 (n=31) | IDS<1 (n=35) | p value |
| Painful colonoscopy rate (%) | 13 (46.4%) | 7 (16.3%) | 0.006 | | 10 (28.6%) | 4 (12.9%) | 0.035 |
| Insertion time^a^ (min) | 8.5(5.7-15.5) | 6.1(4.2-11.4) | 0.047 | | 10.5(6.2-16.8) | 6.9(4.7-10.7) | 0.022 |
| Cecal intubation rate (%) | 27 (96.4%) | 43 (100.0%) | 0.394 | | 29(93.5%) | 34 (97.1%) | 0.597 |
| Abdominal compression (%) | 14 (50.0%) | 9(20.5%) | 0.009 | | 16 (51.6%) | 7 (22.9%) | 0.015 |
| Position change (%) | 17 (60.7%) | 13 (30.2%) |  | | 19 (61.3%) | 12 (34.3%) | 0.028 |

IDS, intubation discomfort score

^a^Values were expressed as medium (range)
