# Supplementary material for: A novel intubation discomfort score to predict painful unsedated colonoscopy
Source: Medicine (Baltimore). 2021 Mar 12;100(10):e24907. doi: 10.1097/MD.0000000000024907 (PMC7969226; doi:10.1097/MD.0000000000024907)
Supplement: Supplemental Digital Content [file medi-100-e24907-s003.docx]

**Supplementary Table 3.** Effect of HAD score on the different colonoscopy

|  | Training cohort (n=345) | | | Validation cohort (n=262) | | |
| --- | --- | --- | --- | --- | --- | --- |
|  | Diagnostic (n=274) | Screening or surveillance  (n=71) | p value | Diagnostic (n=196) | Screening or surveillance  (n=66) | p value |
| HAD score^a^ | 0 (0-4) | 0 (0-3) | 0.338 | 0 (0-3) | 0 (0-4) | 0.709 |
| Moderate and severe Pain (%) | 71(25.9%) | 21(29.6%) | 0.534 | 46(23.5%) | 16(24.2%) | 0.895 |

^a^Values were expressed as medium(range)
